# Youth-centred participatory action approach towards co-created implementation of socially and physically activating environmental interventions in Africa and Europe: the YoPA project study protocol

**DOI:** 10.1136/bmjopen-2024-084657

**Published:** 2024-02-21

**Authors:** Mai J.M. Chinapaw, Leonie H. Klaufus, Adewale L Oyeyemi, Catherine Draper, António L Palmeira, Marlene Nunes Silva, Sara Van Belle, Charlotte S Pawlowski, Jasper Schipperijn, Teatske M Altenburg

**Affiliations:** 1 Public and Occupational Health, Amsterdam UMC Location VUmc, Amsterdam, The Netherlands; 2 Health Behaviours and Chronic Diseases, Amsterdam Public Health Research Institute, Amsterdam, The Netherlands; 3 College of Health Solutions, Arizona State University, Phoenix, Arizona, USA; 4 Department of Physiotherapy, Redeemer's University, Ede, Nigeria; 5 SAMRC Developmental Pathways for Health Research Unit, Faculty of Health Sciences, University of the Witwatersrand, Johannesburg, South Africa; 6 CIDEFES, Universidade Lusófona, Lisboa, Portugal; 7 CIFI2D, Universidade do Porto, Porto, Lisbon; 8 Programa Nacional para a Promoção da Atividade Física, Direcção-Geral da Saúde, Lisboa, Portugal; 9 Department of Public Health, Institute of Tropical Medicine, Antwerpen, Belgium; 10 Department of Sports Science and Clinical Biomechanics, University of Southern Denmark, Odense, Denmark

**Keywords:** adolescents, community-based participatory research, behavior, health equity

## Abstract

**Introduction:**

The majority of adolescents do not meet guidelines for healthy behaviours, posing major risks for developing multiple non-communicable diseases. Unhealthy lifestyles seem more prevalent in urban than rural areas, with the neighbourhood environment as a mediating pathway. How to develop and implement sustainable and effective interventions focused on adolescent health and well-being in urban vulnerable life situations is a key challenge. This paper describes the protocol of a Youth-centred Participatory Action (YoPA) project aiming to tailor, implement, and evaluate social and physical environmental interventions.

**Methods and analysis:**

In diverse urban environments in Denmark, the Netherlands, Nigeria and South Africa, we will engage a dynamic group of 15–20 adolescents (12–19 years) growing up in vulnerable life situations and other key stakeholders (eg, policy makers, urban planners, community leaders) in local co-creation communities. Together with academic researchers and local stakeholders, adolescents will take a leading role in mapping the local system; tailoring; implementing and evaluating interventions during participatory meetings over the course of 3 years. YoPA applies a participatory mixed methods design guided by a novel Systems, User perspectives, Participatory co-creation process, Effects, Reach, Adoption, Implementation and Maintenance framework assessing: (i) the local systems, (ii) user perspectives, (iii) the participatory co-creation process, (iv) effects, (v) reach, (vi) adoption, (vii) implementation and (viii) maintenance of interventions. Through a realist evaluation, YoPA will explore why and how specific outcomes were reached (or not) in each setting (n=800–1000 adolescents in total).

**Ethics and dissemination:**

This study received approval from the ethics committees in Denmark, the Netherlands, Nigeria and South Africa and will be disseminated via various collaborative dissemination activities targeting multiple audiences. We will obtain informed consent from all participants. We envision that our YoPA co-creation approach will serve as a guide for participation of adolescents in vulnerable life situations in implementation of health promotion and urban planning in Europe, Africa and globally.

**Trial registration number:**

NCT06181162.

**Strengths and limitations of this study**

- By introducing teen-centred evidence-informed co-creation—combining a participatory and complex systems approach—Youth-centred Participatory Action (YoPA) proposes a novel approach to the complex challenges of physical inactivity and health inequalities.
- YoPA contributes to theory-building and the evidence base on why and how environmental interventions work (or not) by applying a realist evaluation in diverse, multicountry contexts.
- YoPA fills research gaps in health behaviours and non-communicable diseases within sub-Saharan Africa and the involvement of adolescents in shaping their physical and social environments.
- The complexity of the public health problem and context-specific approach prohibit a randomised controlled trial design.
- Developing actions that change the system is highly ambitious and the involved stakeholders may not have the ability to fully implement the required structural changes within the timeframe of the project.

## Introduction

Insufficient physical activity is associated with many non-communicable diseases (NCDs) and responsible for >5 million deaths worldwide each year.^1^ Public health guidelines recommend at least 60 min/day moderate-to-vigorous physical activity for youth.^2^ An alarming large number of adolescents do not meet these guidelines: at global level, 78% of boys and 85% of girls between the age of 12 and 18 years.^3^ Girls are generally less active than boys^3^ and European adolescents with migrant or ethnic minority backgrounds are generally less active than adolescents from the majority population.^4^ As a result, many adolescents have an increased risk of developing physical inactivity-related NCDs, both physical (eg, obesity, diabetes) and mental (eg, reduced well-being, anxiety, depression).^5–7^ Moreover, recreational activities are an effective coping strategy for many and have a positive effect on reducing stress, especially when physical activity is combined with social support.^8^ The periods of lockdown due to the COVID-19 pandemic exacerbated inequities, and in Europe the percentage of adolescents meeting physical activity recommendations decreased to 9.3% among those aged 9–18 years.^9^ Periods of lockdown were particularly challenging for the most marginalised groups due to urban overcrowding, lack of public open space and lower levels of access to outdoor activities.

Besides the abundant evidence for the benefits of regular participation in physical activity, over the past decade, excessive sedentary behaviour, specifically recreational screen-based behaviour and shortened sleep have gained increased attention as risk factors for adolescents’ health and well-being.^10–12^ Thus, a healthier composition of movement behaviours (ie, physical activity, sedentary behaviour and sleep) throughout the 24 hours of the day has important physical and mental health benefits.^12 13^ Moreover, movement behaviours and their underlying mechanisms interact and might result in a vicious circle of unhealthy behaviours negatively influencing each other.^14^ Physical activity can also be a powerful tool for promoting health equity through community empowerment, mutual social support ensuring affordable access to sport and recreation services.^15^

Recognising the importance and urgency of reducing global levels of insufficient physical activity, WHO member states endorsed a global action plan on physical activity^16^ and agreed to a 15% relative reduction in insufficient physical activity among adolescents by 2030. The International Society of Physical Activity and Health (ISPAH) formulated eight investments that work for physical activity,^17^ which are supported by robust evidence of effectiveness and have worldwide applicability.^18^ Recommended environmental and policy approaches include creation and improvement of access to places for physical activity with informational outreach activities, community-scale and street-scale urban design and land use, a pro-active transport policy and practice and community-wide participatory policies and planning.^19^ Despite these global efforts, most of the evidence on the health benefits of and interventions targeting physical activity is from high-income countries,^20^ or what are increasingly referred to as ‘Minority World’ countries^21^ (as in those countries combined the minority of the world’s population lives). This terminology highlights the absence of representation in research in this field from ‘Majority World’ countries. This is particularly relevant for Africa, which accounts for <1% of global research output even though it makes up 12.5% of the world’s population.^22^ For example, in the field of child development, research from countries in which the majority of the world’s population lives is unacceptably under-represented in most academic journals.^23^

Many interventions targeting adolescents have had disappointing impact, plausibly because they were implemented top-down, adult-driven and insufficiently tailored to the specific context^24^ and thus not addressing the real wishes and needs of adolescents. For example, the beneficial long-term effects of regular physical activity on reducing morbidity and healthcare costs are highly relevant for health professionals and policy makers, while for adolescents the more immediate benefits on well-being, directly or indirectly through mutual social support, and having fun are of relevance. Health professionals increasingly call for greater engagement of young people in the measurement of adolescent health issues as well as the development of appropriate targeted interventions to promote their health.^25^ In programmes that do engage young people, those included are often already confident, articulate and natural leaders.^26^ Instead, engagement of youth growing up in vulnerable life situations (eg, ethnic minorities, living in socially and economically underprivileged neighbourhoods, those with lower educational levels and managing many uncertainties) in implementation of preventive interventions would have greater impact on closing equity gaps in health and well-being. Therefore, in this paper we introduce the novel design and protocol of the EU-funded Youth-centred Participatory Action (YoPA) project.

### The Youth-centred Participatory Action project

Considering the complexity of improving healthy movement behaviours and reducing health inequalities in adolescents, we initiated the YoPA project. The overall aim of YoPA is to optimally tailor, implement and evaluate social and physical environmental interventions using an evidence-informed co-creation approach, for structural improvement in the lifestyle of adolescents (aged 12–18 years) in urban vulnerable life situations in two European and two African cities. YoPA focuses on improving the physical and built environment as well as the social environment considering the importance of friends’ and peers’ influence, and social networks for both physical activity and well-being.^27 28^ Co-creation is a participatory approach of creative and interactive problem-solving among diverse stakeholders with a shared goal and a shared decision-making process, from collaborative problem identification and solution generation to implementation and evaluation.^29^ Through co-creation geared towards adolescent empowerment, we aim to enhance personal and collective agency, and in turn, perceptions of autonomy, which have a direct effect on improving health outcomes.^30^ YoPA aims to contribute to physical activity security which implies that all people, at all times, should have physical and economic access to sufficient, safe and enjoyable physical activity to meet their health needs, and to promote social connectedness and well-being, for an active and healthy life.^20^ In YoPA, we aim to tackle the following four challenges by creating and experimenting with a YoPA approach in four different countries.

### Challenge 1: lifestyles and health inequalities in adolescents continue to worsen

It is crucial to promote healthy movement behaviours in adolescence for multiple reasons: (i) most adolescents fail to meet the three movement behaviour guidelines^31^; (ii) the trend for decreasing physical activity starts in adolescence^32 33^; (iii) screen time increases throughout adolescence^33^; (iv) lifestyle habits, including physical activity and screen time^34 35^ track from adolescence into adulthood; (v) several NCDs have their origins in childhood and adolescence and persist into adulthood^36 37^ thus effective interventions in adolescence can have lifelong and intergenerational health implications; (vi) adolescence is a crucial and vulnerable life transition where adolescents acquire emotional and cognitive abilities for independence. How one navigates this transition depends on available opportunities and resources (eg, family finances to allow school attendance); various systems (eg, transportation, social welfare) and broader societal norms (eg, gender). Adolescents in vulnerable life situations such as living in socio-economic underprivileged areas, minority groups and from low educational and income levels, have less opportunities, and are more at risk for unhealthy lifestyles and worse health outcomes than their mainstream peers.^38^ Living in socially disadvantaged areas doubles adolescents’ risk of engaging in low levels of moderate-to-vigorous physical activity.^39^

### Challenge 2: increasing population density in urban areas limits space for sports and outdoor play

Since 2007, most of the world’s population lives in urban areas with major differences in socio-economic and cultural backgrounds and health.^40^ The way cities are built, and public space is designed impacts many of our conscious and unconscious behavioural choices, acknowledged in ISPAH investment #3 ‘active urban design’. An international study in 14 cities on 5 different continents showed that adults who lived in the most activity-friendly neighbourhoods engaged in 68–89 min more physical activity per week than those living in the least activity-friendly neighbourhoods. Across vastly different cities spread over 10 countries on 5 continents, people living in neighbourhoods with a higher residential density, a more connected street-network, a good public transportation network and more parks were more active than residents living in other neighbourhoods.^41^ Active urban design also positively impacts two other ISPAH investments; #6 equitable access to sport and recreation facilities and amenities, such as parks and urban green spaces, promoting recreational physical activity and #2 active transport through more destinations, shorter distances and better walking, cycling and public transportation infrastructure, thereby generating a potential tipping point for promoting physical activity.^42^ The importance of urban design as well as public and green open spaces in providing a positive, enabling environment for physical activity is well-known.^41 43 44^ However, the increasing population density in urban areas leads to an increased pressure on the public space and in majority countries to an increase in informal settlements and the global privatisation of public space,^45 46^ limiting space for sports and outdoor play.^47^ Scientific evidence supports that the built environment has the potential to affect the long-term health of adolescents by increasing their daily physical activity through a combination of attractive recreational facilities (eg, sport pitches, green spaces, amenities like fresh drinking water).^48 49^ Nonetheless, current urban environments serve adults and young children better than adolescents.^50^ Nonetheless, youth have different access to power than the professionals who plan the public spaces of their neighbourhood. Especially girls’ access to public space adapted to their specific needs could be improved.^51^ To increase effectiveness of socio-environmental interventions, there is a need for studies that consider the perceptions of different intersectional groups of adolescents (eg, boys and girls with varying sociocultural backgrounds) in designing an attractive environment or public space.^52^

### Challenge 3: traditional individual-level behavioural interventions are less sustainable

Physical inactivity is a complex public health problem with multiple interacting influences and feedback loops embedded in social, cultural and physical systems.^53^ Such complex problems require multiple, upstream and downstream, embedded population-level actions that favourably contribute to reshaping nested systems.^54^ Effective approaches to tackling physical inactivity will thus require multiple concurrent strategies and actions to be implemented across settings and sectors. However, to date, physical activity interventions have primarily focused on isolated causes and linear relationships with individual-level health outcomes rather than a systems approach that considers the links, feedback loops and interactions among elements within the bigger picture.^55^ For example, most physical activity interventions have primarily relied on educational and information-based programmes targeting the individual with little consideration of the relational and social (eg, peers, role models, gatekeepers) and physical environments (eg, accessibility of parks, walkability, adequate lighting, safety) that have a major enabling or hindering influence on health behaviours.

### Challenge 4: top-down implemented, one-size-fits-all interventions are ineffective

Health research frequently addresses questions and outcomes that are of limited relevance to healthcare practitioners, patients and other end-users, resulting in considerable research waste.^56^ Hence, most top-down, adult-driven, standardised interventions have had limited adoption and impact.^24^ Citizen participation in the form of youth-centred, evidence-informed co-creation of interventions tailored to local contexts helps to prevent misalignment of priorities between researchers and stakeholders on the one hand and misalignment of interventions with local contexts on the other. Engaging adolescents as critical agents of social and political change is necessary for building inclusive democratic societies, which can result in more effective and youth-friendly health promotion.^57 58^ Currently, adolescents increasingly participate in public health research; however, participation is generally limited to consultation and adolescents are rarely involved in the decision-making process, which is essential to becoming empowered and gain personal and collective agency to take action to improve their life situation.^59–62^ Several studies on youth participation in policy making have demonstrated that young people are sharp analysts of their settings and creative producers of ideas for planning, but authorities are reluctant to expand their top-down, expert-based mode of urban planning and health policy making to include young people.^63 64^

Here, we present the protocol of the YoPA project including the design, theoretical and evaluation framework.

## Methods and analysis

### Design

**Figure 1:**
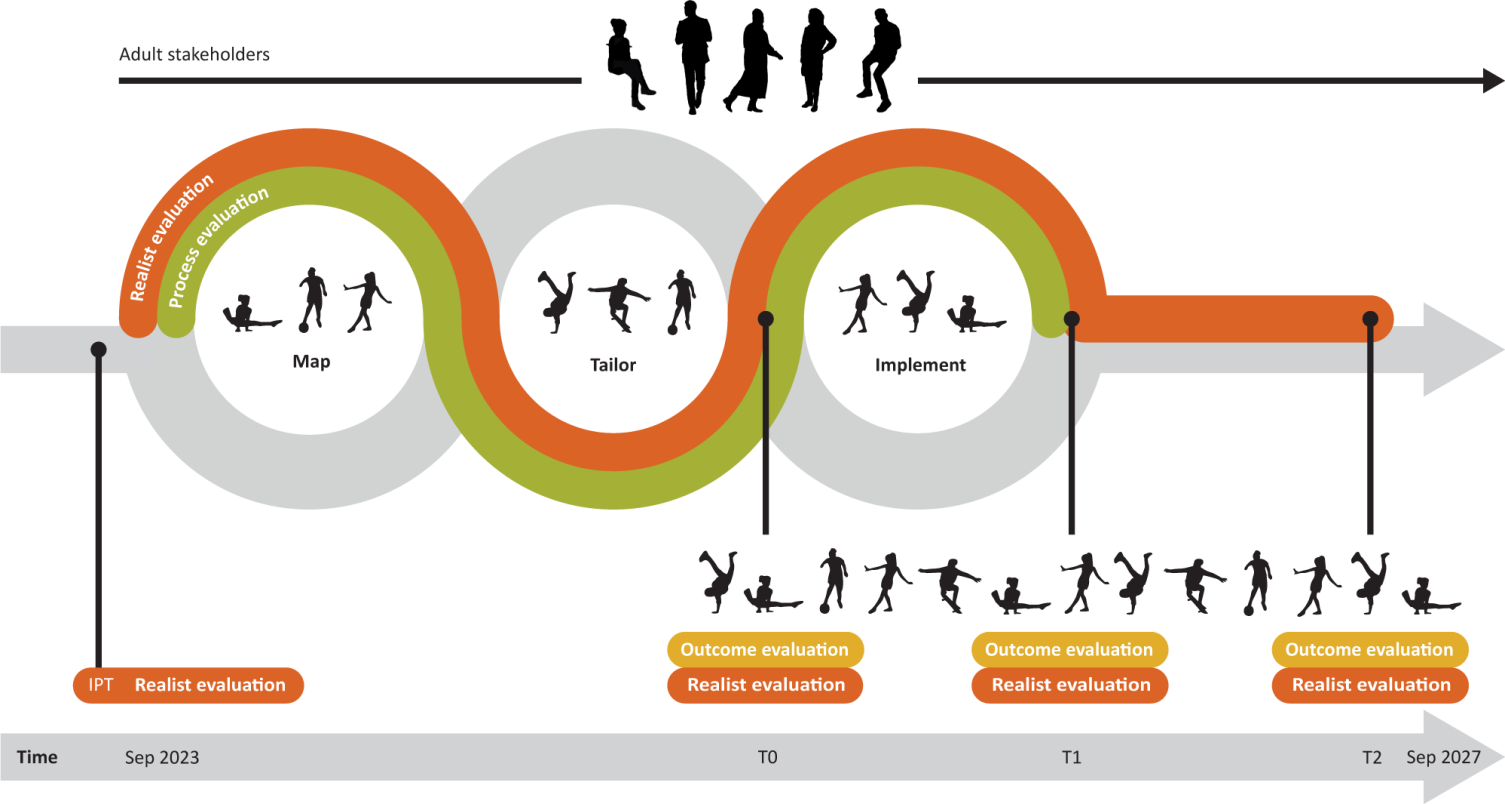
Youth-centred Participatory Action approach visualising the engagement of adolescent researchers, community adolescents and adult stakeholders. IPT, initial programme theory.

YoPA combines the flexible and adaptive participatory action research with a rigorous practical protocol and evaluation framework as well as scientific evidence with systematically produced local knowledge, that is, knowledge that is rooted in experience in a particular social context. Figure 1 presents the five phases of the YoPA approach, where engagement of stakeholders and evaluation continue throughout the project. We use a participatory,^65^ mixed-methods,^66^ comparative approach^67 68^ to comprehensively examine a broad range of evaluation questions such as whether, how and why interventions contribute to system change; how this evidence can be generalised and subsequently adapted to specific contexts, intended and unintended consequences of implemented interventions as well as potential working mechanisms and interactions with the local context. Using our novel Systems, User perspectives, Participatory co-creation process, Effects, Reach, Adoption, Implementation and Maintenance (SUPER-AIM) framework (table 1), we will evaluate both the participatory co-creation process as well as the process and outcomes of implementing holistic, systemic interventions in the four study sites: Aalborg in Denmark, Amsterdam in the Netherlands, Osogbo in Nigeria and Soweto in South Africa.

**Table 1:** The YoPA SUPER-AIM evaluation framework

Component and definition	Outcome	Methods
*Systems*—identification of the drivers of unhealthy movement behaviours at multiple levels of the system including linkages, relationships, feedback loops and interactions among system parts	Maps of the local system and its stakeholders, displaying knowledge gaps, leverage points for interventions and insights Overview of both intended and emergent outcomes of interventions across various levels, interactions with the local context and adaptation of interventions	Developing local system maps based on, for example, Group Model Building,^88^ Social Network Analysis.^89^ Ripple Effects Mapping^90 91^: in several group sessions, different key stakeholders participate to provide their perspective on the outcomes (appreciative inquiry) and collaboratively explore the contribution of the implemented interventions to these outcomes in mind maps. This provides practice-based knowledge about effective principles as well as the broader impact of the interventions.
*User perspectives*—identification of the user perspective on implemented interventions, for example, on the attractiveness and acceptability	Accessibility, acceptability and adaptations of interventions, for example, perceived physical activity friendliness, perceived inclusiveness of interventions, perceived safety and fear of crime; satisfaction with interventions and use of interventions	Participant observation and in-depth formal and informal interviews with adolescents, for example, using photo-diaries,^92^ go-along interviews,^93 94^ neighbourhood audit,^95^ focus group interviews.^96 97^
*Participatory co-creation process*—identification of important barriers and facilitators of the participatory co-creation process	Adolescents’ motivations to participate in the project Satisfaction with the co-creation process among involved stakeholders* Mechanisms underlying co-creation	Participatory observations, focus group interviews, reflection scheme after each co-creation meeting. Online satisfaction measurement, focus group interviews. In-depth focus group interviews^98^ with project team; realist context-mechanism-outcome causal analysis.
*Effects*—identification of desired outcomes among the adolescents. If necessary, measures of locally defined impact will be added to examine factors of greatest interest to local stakeholders	Well-being Personal and collective agency 24 hour movement behaviours (physical activity, sedentary behaviour, sleep)	EPOCH measure of adolescent well-being.^99^ GEAS survey on freedom of movement, voice, behavioural control and decision making.^100^ Accelerometers, self-report and systematic observation (adapted SOPLAY^96^/SOPARC^97^).
*Reach*—adolescents whose behaviours and well-being we aim to benefit: (1) co-creation participants; (2) users of interventions; (3) adolescent citizens in the selected communities	Characteristics of adolescents	Focus group interviews with co-creation participants. Systematic observations of intervention users. Existing databases (eg, from municipality) and survey data of adolescent citizens in the selected communities.
*Adoption*—identification of engagement and commitment with (1) implemented interventions; (2) teen-centred co-creation	Engagement and commitment of relevant stakeholders*	Focus group interviews.
*Implementation*—identification of adaptations, potential barriers and facilitators of implementation	Satisfaction with implementation of youth-centred co-creation among involved stakeholders* Number, type and quality of implemented interventions Satisfaction with implementation of interventions among involved stakeholders* Costs of intervention implementation	Participatory observations and focus group interviews. Calculation of the resources needed to implement the interventions using microcosting.^85^
*Maintenance*—identification of sustained use of (1) implemented interventions; (2) teen-centred co-creation	Sustained use of interventions Sustained use of youth-centred co-creation in the communities	Systematic observation (eg, adapted SOPLAY^96^/SOPARC^97^). Focus group interviews.

*Involved stakeholders: for example, adolescents, public health professionals, urban planners/designers, policy makers.

EPOCH, Engagement, Perseverance, Optimism, Connectedness, and Happiness; GEAS, Global Early Adolescent Study; SOPARC, System for Observing Play and Recreation in Communities; SOPLAY, System for Observing Play and Leisure Activity in Youth; SUPER-AIM, Systems, User perspectives, Participatory co-creation process, Effects, Reach, Adoption, Implementation and Maintenance; YoPA, Youth-centred Participatory Action.

### Theories and paradigms

The central paradigm in YoPA is participatory action research: a collaborative, iterative, often open-ended and unpredictable approach, which prioritises the expertise of those experiencing a social issue and uses systematic research methodologies to generate new insights.^65^ In YoPA, we use the six building blocks for designing a participatory action research project proposed by Cornish *et al* 65: (i) building relationships, (ii) establishing working practices, (iii) establishing a common understanding of the issue, (iv) observing, gathering and generating materials, (v) collaborative data analysis and (vi) planning and taking action. A key benefit of participatory action research is empowerment by enabling participants to have a voice and contribute to knowledge production.^69 70^ Empowerment theory is a conceptual framework for understanding the enhancement of positive youth development by engaging youth in developing confidence, skills and behavioural strategies to achieve self-identified goals.^71 72^ Empowerment includes three components: (1) intrapersonal, including beliefs regarding control and confidence; (2) interactional, including critical awareness of driving forces and understanding of the actions and resources needed for the desired change and (3) behavioural, referring to actions to make the desired changes.^71^ A second paradigm in YoPA is a systems-approach that considers the links, feedback loops and interactions among elements within the bigger picture.^55^ We start with studying and understanding the local context. Next, we aim to develop and implement interventions, which we consider as a complex of actions that favourably contribute to reshaping the system dynamics.^14^

### The YoPA co-creation protocol

We will start with collaboratively developing one overall YoPA co-creation protocol together with the local researchers from all four study sites. The YoPA co-creation protocol aims at high-quality co-creation (i) based on state-of-the-art science-based and practice-based evidence and theory; (ii) tailored to the local context, including the local needs and preferences of adolescents; (iii) acceptable and feasible for local stakeholders responsible for implementation. This protocol ensures a systematic, evidence-based and theory-based application of co-creation leaving space for adaptation to each local context. The overall co-creation protocol will include building an infrastructure for continuous capacity building for adolescents, as well as local stakeholders to stimulate participatory thinking, active engagement, equal collaboration and training in research and other relevant skills. This protocol will describe how to apply youth-centred co-creation including recruitment and all methods for capacity building and peer research. We will organise training for local facilitators of the youth-centred co-creation process, as well as for key stakeholders to stimulate their active contribution to the co-creation process. Academic researchers bring in their state-of-the-art scientific knowledge and experience with developing evidence-based interventions while adolescent researchers bring in their lived experience. In YoPA, we aim to develop academic and adolescent researchers’ collective agency, by building their capacities for collaboration, peer-research and intervention development. Collaborating with other key stakeholders from multiple sectors in the system will gain a deeper understanding of the complex system and thereby contribute to more holistic and contextually relevant interventions.

### Engaging local YoPA communities

We will engage four local co-creation communities, two communities in minority countries (Denmark and the Netherlands) and two in majority countries (Nigeria and South Africa). In each community, a dynamic group of 15–20 adolescents will be recruited to participate as co-researchers in local co-creation groups facilitated by an academic researcher. Recruitment will take place through diverse channels and settings including schools, local community centres, youth clubs, religious meeting places and other relevant settings where adolescents with diverse backgrounds meet. We will use a purposive sampling method tailored to each local context (eg, social media, flyers) in collaboration with local non-governmental organisations and other community stakeholders. By ensuring safe spaces, skilled facilitators and capacity building, adolescents in local co-creation groups will be encouraged to actively engage and contribute to the co-creation process. We will conduct stakeholder analyses to identify and recruit other key stakeholders (eg, existing community-based organisations and local authorities with a shared agenda), who will be invited to actively contribute to the co-creation process by joining meetings of the local co-creation groups. The co-creation process will take place during regular participatory meetings with adolescents facilitated by an academic researcher over the course of 3 years. To maximise chances of sustained commitment, we will collaborate with local community groups organised around health advocacy, sports, music or social activity. We will emphasise social inclusion by involving adolescents of different genders and backgrounds.

### Mapping the local context

To ensure YoPA will address questions and outcomes that are most relevant to the local communities, thereby promoting uptake and sustainability of the interventions, we will start with mapping the local context by an audit and environmental scan of selected communities to identify local needs and priorities using various state-of-the-art participatory methods, for example, photovoice,^73^ community mapping^74^ and neighbourhood walks.^75^ To explore the local communities at multiple levels, that is, including linkages, relationships, feedback loops and interactions, we will use systems methods such as group model building^76^ and social network analysis.^77^ We will use Causal Loop Diagrams as a tool to explore the multiple, interacting feedback loops operating in the existing local system. Such Causal Loop Diagrams create a dynamic, holistic view of the existing system, including intended and unintended potential consequences, and the ways in which interventions in one setting, such as home or school, might be influenced by the interactions with other settings, such as macroeconomic and urban systems, for example, public space.^14 78^ In bringing together key stakeholders (locally, nationally or internationally) to understand the root causes of unhealthy movement behaviours, a systems approach enables each stakeholder to see where they fit within a bigger picture.^17^ To ensure results align with the perspectives of the wider community, emerging findings will be shared with community representatives for them to critically examine and contribute. For this step, we may use structured interview matrix^79^—a community-based research method that allows large groups (up to 40 participants) to discuss directions for future developments and priorities in an iterative, structured and transparent process—and multicriteria decision-making matrices,^80^ to weigh all collected data in a transparent way. Each local system map will include an agreed set of priorities for holistic, systemic interventions in each local community.

### Selection, tailoring and implementing evidence-informed interventions

Based on the local system maps, the best matching evidence-based interventions will be selected from (i) local youth-led knowledge, (ii) ISPAH’s eight investments that work for physical activity; (iii) literature reviews conducted by the academic researchers and (iv) other relevant (local) literature and databases including evidence-based interventions. For each of the selected interventions, we will develop an intervention theory to help identify key working mechanisms, salient context conditions and relevant additional outcomes. The intervention theories will be grounded in existing evidence and empirically tested in the local contexts. The selected interventions will be aligned with local priorities and existing strategic plans where possible, based on the local system maps and meetings with key stakeholders, to obtain support and ensure feasibility, sustainability and resources for the implementation. Key considerations for our settings are safety and crime (especially for adolescent girls), limited infrastructure and resources and transport challenges.

### Evaluate interventions using the SUPER-AIM framework

The YoPA evaluation will take a systems perspective, aiming to evaluate a range of outcomes, associated processes and their dynamic inter-relationships using interrupted time series methods as one of the strongest quasi-experimental research designs.^81^ Table 1 describes the specific outcomes, samples and proposed methods for each component of our SUPER-AIM framework. Together with the local co-creation communities, we will select and/or modify the most appropriate methods that allow the collection of quantitative and qualitative data at all system levels, including measures of the process and outcomes of the co-creation and implementation of interventions. Process data will be collected continuously from the start of the co-creation process. Outcome data will be collected before and 6 months after implementation of interventions as well 6–12 months later depending on the local situation. For the outcome evaluation, we aim to recruit 200–250 adolescents in each local community. Training of (adolescent) data collectors for collecting data in the four communities will follow the ‘train-the-trainer’ principle: one meeting will be organised to train the researchers responsible for data collection in their country, who will subsequently train local (adolescent) data collectors. As there is a lack of evidence on the application of youth-centred co-creation in vulnerable settings in both majority and minority countries, we aim to better understand the mechanisms underlying co-creation through personal and collective agency in each of the settings with the help of a realist evaluation.^82^ Next to evaluating the outcomes of interventions, realist evaluation aims to understand why and how specific outcomes were reached in each setting and thus contributes to building the theory base on why interventions work (or not), and for whom, in a range of settings. Collaborating and sharing experiences across the four co-creation sites through online meetings, exchanges and joint analyses may help to generalise findings.

### Analyses

Data collected by adolescent-researchers throughout the co-creation process will be analysed using the best available and accessible techniques with options for facilitated co-researcher involvement. The selected methods should be engaging to the co-researchers, suited to answering their research questions and supported by a skilled academic researcher. Following data cleaning and data processing, we will analyse the outcomes of the implemented interventions, as well as the dynamics underlying these, combining and comparing data from the four study sites. We will conduct analyses of a combination of quantitative (eg, sensor-based behavioural data) as well as qualitative (eg, interviews and user-generated data) data.^83^ Quantitative data will be analysed using appropriate techniques (eg, multilevel modelling appropriate for individual-level data nested within communities). Qualitative data will be summarised and subsequently analysed using open and axial coding by two independent researchers. Intersectionality references the critical insight that race, class, gender, sexuality, ethnicity, nation, ability and age operate not as unitary, mutually exclusive entities, but as reciprocally constructing phenomena that in turn shape complex social and health inequalities.^84^ In both quantitative and qualitative analyses, we will apply different kinds of intersectional analyses including relevant categories such as gender, age, education and ethnicity.

In the social network analysis, we will focus on the relationships among relevant ‘actors’ when mapping the local setting including persons, organisations and locations to understand the inter-relations and impacts of factors at different levels—from individual-level factors to environments and policies. We will use this knowledge to identify leverage points for interventions. We will integrate realist evaluation^82^ in the process evaluation to better understand which mechanisms contributed to the observed outcomes, for example, how the achievement of individual and collective agency leads to empowerment, and under which conditions. Additionally, we will provide a tested and refined intervention theory on the application of youth-centred co-creation in vulnerable settings, focusing on social mechanisms potentially to be triggered (trust, reciprocity, neighbourhood solidarity, personal and collective agency, leadership) in a range of context conditions (typology of settings: socially cohesive long time residing migrant communities, less cohesive transient migrant communities, diverse communities, partially gentrified, etc). We will develop a plausible causal explanation, focusing also on counteracting or unintended consequences. These findings will be further synthesised into a refined intervention theory that can be used for future similar interventions and can be tested in other settings. To analyse the costs of implementation, we will use microcosting reflecting actual resource use and economic costs by collecting data on resources used and the unit costs of those resources following guidelines and checklists for conducting and reporting microcosting studies.^85^

### Patient and public involvement

Involvement of youth and other relevant stakeholders is a key element of the YoPA project. Together with academic researchers and local stakeholders, adolescents will take a leading role in the co-creation process running over the course of 3 years (see also ‘Engaging local YoPA communities’ section). Recruitment of adolescents for the local co-creation communities started in October 2023 in Denmark and in January 2024 in all other countries. Data collection will continue until December 2026.

## Ethics and dissemination

Ethical considerations are fundamental throughout the YoPA project. In YoPA, we will encourage an emphasis on inclusive practices, mutual respect, continuous dialogue and reflexivity, shared decision-making and collaborative action. Each adolescent participating in the youth-centred co-creation or any aspect of the evaluation, and where relevant also one of their parents, will sign informed consent before participating in the study, verifying that they understood the involvement and agree to data collection. We will develop attractive, age-adapted and easy-to-understand information (eg, brochures, videos) explaining the purpose of involvement, the nature of data collection, the potential burden (eg, time investment), the right to access their own data, how data will be processed and protected and how confidentiality will be maintained. Where possible, we will make datasets generated and/or analysed during the YoPA project available in the Open Science Framework repository. Not all data can be made public in order to protect participants’ confidentiality. Participation is entirely voluntary, and participants can choose to withdraw at any time without consequences. The Research Ethics committees of the four local institutions approved the protocol for the YoPA project: Amsterdam UMC Medical Ethical Committee, Netherlands (2023.0670), the Redeemer’s University, Nigeria (2023.060), the University of Southern Denmark Research Ethics Committee, Denmark (Case no 23/47839, REC ID 408), the Human Research Ethics Committee (Medical) at the University of the Witwatersrand, South-Africa (reference: M230721).

**Figure 2:**
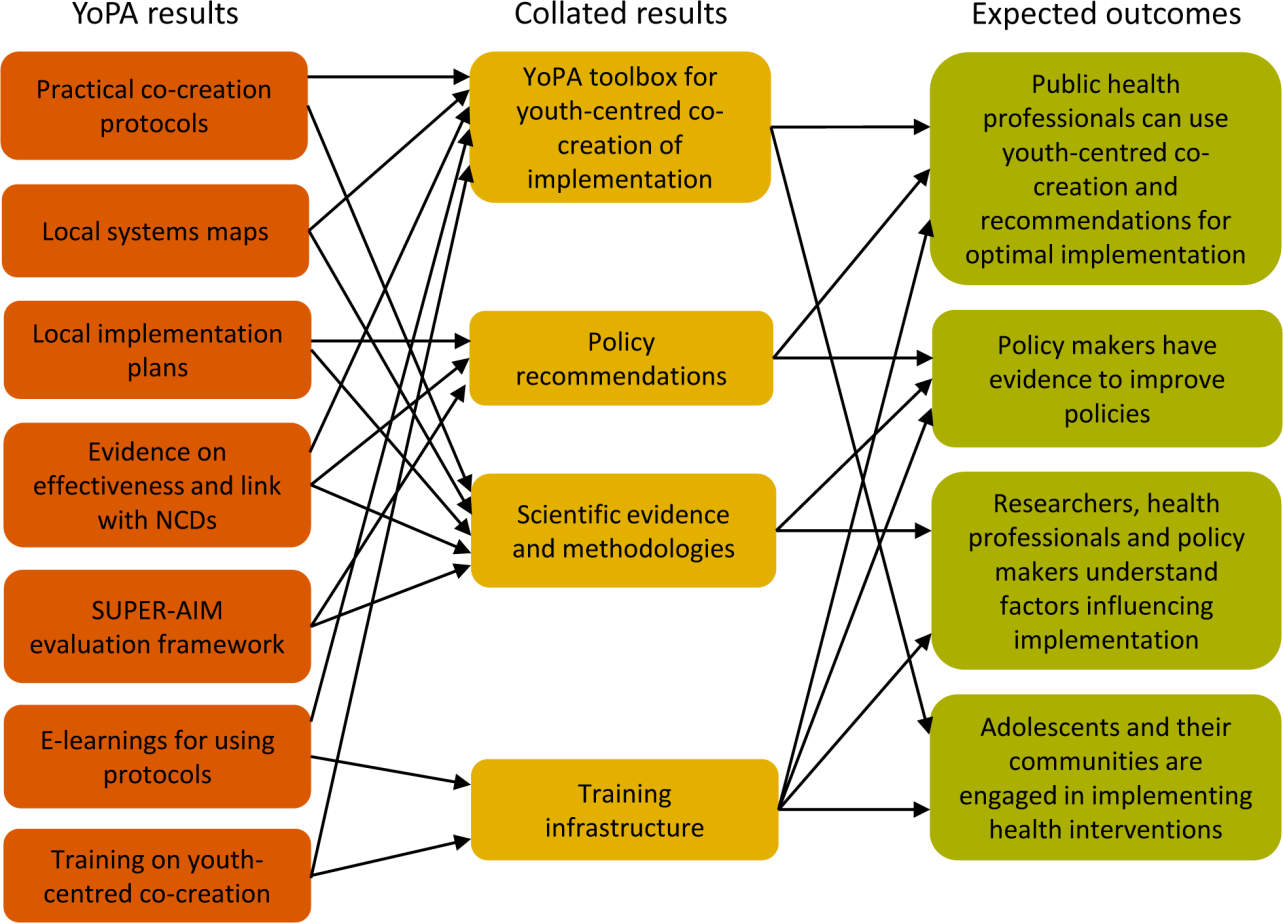
Communication, dissemination and impact plan of the YoPA project. NCD, non-communicable disease; SUPER-AIM, Systems, User perspectives, Participatory co-creation process, Effects, Reach, Adoption, Implementation and Maintenance.

To enhance the communication, dissemination and impact of YoPA, we have developed a comprehensive plan (figure 2) that includes a well-defined strategy, clear objectives with measurable key results and various tools designed to amplify the project’s impact. Effective communication and (community) dialogue is crucial for raising public awareness about the importance of healthy movement behaviours in preventing NCDs and promoting youth-centred co-creation of intervention customization and implementation. This will enhance the visibility of the YoPA project among various stakeholders, for example, through the project website (https://www.yopa-project.eu/). Collaborative dissemination activities target scientific, stakeholder, policymaker and wider audiences aiming to promote youth-centred co-creation for healthy movement behaviours and NCD prevention tailored to local communities. YoPA is committed to continued project results through a sustainable dissemination and impact strategy. Additionally, we aim to build capacities among local partners and universities for ongoing local co-creation research and community collaboration. We will make all educational and training materials, practical protocols and successful local intervention examples available in the YoPA toolbox. The YoPA approach will be shared through a licensed train-the-trainer programme for effective dissemination through diverse channels. By actively engaging stakeholders in training sessions, we aim to promote the benefits of co-creation and inspire more effective action towards promoting health across society.

## Discussion

YoPA will contribute to health equity by specifically focussing on improving the social and physical environment of adolescents in vulnerable urban life situations. Evaluating the effectiveness of such socio-environmental interventions across heterogeneous local contexts, co-creation communities and interventions is challenging as these will result in different ‘intervention theories’ or scenarios, on how systems-oriented interventions are expected to work in their respective contexts. Describing and testing plausible mechanisms of how interventions are expected to work at multiple levels and for a range of actors (in nested systems), is important for strengthening robust causal inference but also for credibility towards policy and practice.^86^ Traditional designs and analysis methods are not appropriate for studying complex systems as they lack the ability to measure and understand contextual including socio-ecological effects as well as the dynamic properties of complex adaptive systems,^77^ including unintended effects on other parts of the system.^87^ Therefore, we introduce our novel SUPER-AIM framework, incorporating crucial data explaining if, how, why and in which settings the implemented interventions will favourably contribute to reshaping local systems.

A better understanding of how culture and structure impacts the co-creation process and interventions implemented in the four selected communities in YoPA benefits knowledge exchange between the different settings. Furthermore, YoPA goes beyond addressing a research gap in physical activity and health research in sub-Saharan Africa; it takes an approach to considering context in a robust and meaningful way that fully accounts for competing priorities in African settings.^20^ Currently, there is a lack of systematic and practical protocols guiding the application of co-creation for tailoring evidence-informed interventions to specific contexts, and subsequently evaluating them together with adolescents and other key stakeholders. To fill this gap, we will develop a YoPA toolbox, making all materials and training on the youth-centred co-creation for tailoring and implementation of evidence-informed interventions available through the YoPA website (yopa-project.eu), both during its development and its final form. Once results from the process, outcome and realist evaluations start to come in, more formalised guidelines for the use of the toolbox, as well as policy recommendations for the implementation of similar co-creation processes will be developed and become part of the toolbox, targeted at researchers, public health and urban planning practitioners, local authorities, policy makers, grassroots/community-based organisations and citizens.

Limitations of our study could be the lack of a controlled design and the challenge to instigate and measure sustainable system change as this cannot be externally directed, but occurs as a result of the self-organising interactions and relationships within the system. The complexity of the public health problem and context-specific approach prohibit a randomised controlled trial design. Instead, in YoPA we focus on identifying working mechanisms and detailed documentation using a mixed methods design.

By establishing an infrastructure for youth-centred co-creation including capacity building, mentoring and with active engagement of adolescent health advocates and leaders, YoPA aims to nurture sustainable implementation of adolescent-responsive preventive interventions tailored to the local context, improving their agency, 24-hour movement behaviours and well-being, with the purpose of halting the rise in NCDs and associated healthcare costs. We envision that our YoPA youth-centred co-creation approach will serve as a guide for participation of adolescents in vulnerable life situations in implementation of health promotion in Europe, Africa and globally.

## Supplementary Material

Reviewer comments

Author's manuscript

## References

[1] Lee IM , Shiroma EJ , Lobelo F , et al . Effect of physical inactivity on major non-communicable diseases worldwide: an analysis of burden of disease and life expectancy. Lancet 2012;380:219–29. 10.1016/S0140-6736(12)61031-9 22818936 PMC3645500

[2] Bull FC , Al-Ansari SS , Biddle S , et al . World Health Organization 2020 guidelines on physical activity and sedentary behaviour. Br J Sports Med 2020;54:1451–62. 10.1136/bjsports-2020-102955 33239350 PMC7719906

[3] Guthold R , Stevens GA , Riley LM , et al . Global trends in insufficient physical activity among adolescents: a pooled analysis of 298 population-based surveys with 1.6 million participants. Lancet Child Adolesc Health 2020;4:23–35. 10.1016/S2352-4642(19)30323-2 31761562 PMC6919336

[4] Brug J , van Stralen MM , Chinapaw MJM , et al . Differences in weight status and energy-balance related behaviours according to ethnic background among adolescents in seven countries in Europe: the ENERGY-project. Pediatr Obes 2012;7:399–411. 10.1111/j.2047-6310.2012.00067.x 22730265

[5] van Sluijs EMF , Ekelund U , Crochemore-Silva I , et al . Physical activity behaviours in adolescence: current evidence and opportunities for intervention. Lancet 2021;398:429–42. 10.1016/S0140-6736(21)01259-9 34302767 PMC7612669

[6] Akseer N , Mehta S , Wigle J , et al . Non-communicable diseases among adolescents: current status, determinants, interventions and policies. BMC Public Health 2020;20. 10.1186/s12889-020-09988-5 PMC7734741 33317507

[7] Rodriguez-Ayllon M , Cadenas-Sánchez C , Estévez-López F , et al . Role of physical activity and sedentary behavior in the mental health of preschoolers, children and adolescents: a systematic review and meta-analysis. Sports Med 2019;49:1383–410. 10.1007/s40279-019-01099-5 30993594

[8] Biddle SJH , Asare M . Physical activity and mental health in children and adolescents: a review of reviews. Br J Sports Med 2011;45:886–95. 10.1136/bjsports-2011-090185 21807669

[9] Kovacs VA , Brandes M , Suesse T , et al . Are we underestimating the impact of COVID-19 on children’s physical activity in Europe? - a study of 24,302 children. Eur J Public Health 2022. 10.1093/eurpub/ckac003 PMC9159340 35022680

[10] Hjorth MF , Chaput J-P , Damsgaard CT , et al . Low physical activity level and short sleep duration are associated with an increased cardio-metabolic risk profile: a longitudinal study in 8-11 year old Danish children. PLoS ONE 2014;9:e104677. 10.1371/journal.pone.0104677 25102157 PMC4125285

[11] Chastin SFM , Palarea-Albaladejo J , Dontje ML , et al . Combined effects of time spent in physical activity, sedentary behaviors and sleep on obesity and cardio-metabolic health markers: a novel compositional data analysis approach. PLoS One 2015;10:e0139984. 10.1371/journal.pone.0139984 26461112 PMC4604082

[12] Rollo S , Antsygina O , Tremblay MS . The whole day matters: understanding 24-hour movement guideline adherence and relationships with health indicators across the LifeSpan. J Sport Health Sci 2020;9:493–510. 10.1016/j.jshs.2020.07.004 32711156 PMC7749249

[13] Roenneberg T . Chronobiology: the human sleep project. Nature 2013;498:427–8. 10.1038/498427a 23803826

[14] Waterlander WE , Singh A , Altenburg T , et al . Understanding obesity-related behaviors in youth from a systems dynamics perspective: the use of causal loop diagrams. Obes Rev 2021;22:e13185. 10.1111/obr.13185 33369045 PMC8243923

[15] Laverack G . Improving health outcomes through community empowerment: a review of the literature. J Health Popul Nutr 2006;24:113–20. 16796158

[16] World Health Organization . Global action plan on physical activity 2018-2030: more active people for a healthier world. World Health Organization; 2019.

[17] Milton K , Cavill N , Chalkley A , et al . Eight investments that work for physical activity. J Phys Act Health 2021;18:625–30. 10.1123/jpah.2021-0112 33984836

[18] World Health Organization . Tackling NCDs: 'best BUYS' and other recommended interventions for the prevention and control of Noncommunicable diseases. 2017. Available: https://apps.who.int/iris/handle/10665/259232

[19] Heath GW , Parra DC , Sarmiento OL , et al . Evidence-based intervention in physical activity: lessons from around the world. Lancet 2012;380:272–81. 10.1016/S0140-6736(12)60816-2 22818939 PMC4978123

[20] Lambert EV , Kolbe-Alexander T , Adlakha D , et al . Making the case for 'physical activity security': the 2020 WHO guidelines on physical activity and sedentary behaviour from a global South perspective. Br J Sports Med 2020;54:1447–8. 10.1136/bjsports-2020-103524 33239348

[21] Khan T , Abimbola S , Kyobutungi C , et al . How we classify countries and people and why it matters. BMJ Glob Health 2022;7:e009704. 10.1136/bmjgh-2022-009704 PMC9185389 35672117

[22] Marincola E , Kariuki T . Quality research in Africa and why it is important. ACS Omega 2020;5:24155–7. 10.1021/acsomega.0c04327 33015430 PMC7528179

[23] Draper CE , Barnett LM , Cook CJ , et al . Publishing child development research from around the world: an unfair playing field resulting in most of the world’s child population under-represented in research. Infant Child Dev 2023;32. 10.1002/icd.2375

[24] Gibbs A , Campbell C , Maimane S , et al . Mismatches between youth aspirations and participatory HIV/AIDS programmes in South Africa. Afr J AIDS Res 2010;9:153–63. 10.2989/16085906.2010.517482 25860524

[25] Sawyer SM , Afifi RA , Bearinger LH , et al . Adolescence: a foundation for future health. Lancet 2012;379:1630–40. 10.1016/S0140-6736(12)60072-5 22538178

[26] Villa-Torres L , Svanemyr J . Ensuring youth’s right to participation and promotion of youth leadership in the development of sexual and reproductive health policies and programs. J Adolesc Health 2015;56:S51–7. 10.1016/j.jadohealth.2014.07.022 25528979

[27] Maturo CC , Cunningham SA . Influence of friends on children’s physical activity: a review. Am J Public Health 2013;103:e23–38. 10.2105/AJPH.2013.301366 PMC3682627 23678914

[28] Lamblin M , Murawski C , Whittle S , et al . Social connectedness, mental health and the adolescent brain. Neurosci Biobehav Rev 2017;80:57–68. 10.1016/j.neubiorev.2017.05.010 28506925

[29] Vargas C , Whelan J , Brimblecombe J , et al . Co-creation, co-design, co-production for public health - a perspective on definition and distinctions. Public Health Res Pract 2022;32:3222211. 10.17061/phrp3222211 35702744

[30] Wallerstein N . Powerlessness, empowerment, and health: implications for health promotion programs. Am J Health Promot 1992;6:197–205. 10.4278/0890-1171-6.3.197 10146784

[31] Tapia-Serrano MA , Sevil-Serrano J , Sánchez-Miguel PA , et al . Prevalence of meeting 24-hour movement guidelines from pre-school to adolescence: a systematic review and meta-analysis including 387,437 participants and 23 countries. J Sport Health Sci 2022;11:427–37. 10.1016/j.jshs.2022.01.005 35066216 PMC9338333

[32] Kalman M , Inchley J , Sigmundova D , et al . Secular trends in moderate-to-vigorous physical activity in 32 countries from 2002 to 2010: a cross-national perspective. Eur J Public Health 2015;25 Suppl 2:37–40. 10.1093/eurpub/ckv024 25805785

[33] Marques A , Loureiro N , Avelar-Rosa B , et al . Adolescents' healthy lifestyle. Jornal de Pediatria 2020;96:217–24. 10.1016/j.jped.2018.09.002 30393010 PMC9432147

[34] Jones RA , Hinkley T , Okely AD , et al . Tracking physical activity and sedentary behavior in childhood: a systematic review. Am J Prev Med 2013;44:651–8. 10.1016/j.amepre.2013.03.001 23683983

[35] Telama R , Yang X , Leskinen E , et al . Tracking of physical activity from early childhood through youth into adulthood. Med Sci Sports Exerc 2014;46:955–62. 10.1249/MSS.0000000000000181 24121247

[36] Singh AS , Mulder C , Twisk JWR , et al . Tracking of childhood overweight into adulthood: a systematic review of the literature. Obes Rev 2008;9:474–88. 10.1111/j.1467-789X.2008.00475.x 18331423

[37] Yan Y , Hou D , Zhao X , et al . Childhood adiposity and nonalcoholic fatty liver disease in adulthood. Pediatrics 2017;139:e20162738. 10.1542/peds.2016-2738 28356335 PMC5369672

[38] Patton GC , Sawyer SM , Santelli JS , et al . Our future: a lancet commission on adolescent health and wellbeing. Lancet 2016;387:2423–78. 10.1016/S0140-6736(16)00579-1 27174304 PMC5832967

[39] Heath GW , Bilderback J . Grow healthy together: effects of policy and environmental interventions on physical activity among urban children and youth. J Phys Act Health 2019;16:172–6. 10.1123/jpah.2018-0026 30626275

[40] Oosterlynck S , Verschraegen G , Kempen R . Divercities: understanding super-diversity in deprived and mixed neighbourhoods; 2018. 1–250.

[41] Sallis JF , Cerin E , Conway TL , et al . Physical activity in relation to urban environments in 14 cities worldwide: a cross-sectional study. Lancet 2016;387:2207–17. 10.1016/S0140-6736(15)01284-2 27045735 PMC10833440

[42] Wijntuin P , Koster M . Dutch-Moroccan girls navigating public space: wandering as an everyday spatial practice. Space Cult 2019;22:280–93. 10.1177/1206331218794603

[43] Giles-Corti B , Broomhall MH , Knuiman M , et al . Increasing walking: how important is distance to, attractiveness, and size of public open space. Am J Prev Med 2005;28:169–76. 10.1016/j.amepre.2004.10.018 15694525

[44] Giles-Corti B , Vernez-Moudon A , Reis R , et al . City planning and population health: a global challenge. Lancet 2016;388:2912–24. 10.1016/S0140-6736(16)30066-6 27671668

[45] Agyemang F . Privatization of public spaces and its impact on the socio-political and spatial landscapes of the Cape town central city improvement district (CCCID). SSRN Journal 2017. 10.2139/ssrn.3153812

[46] Ware C . A tale of two cities: public space development in Nigeria. 2021. Available: https://worldlandscapearchitect.com/a-tale-of-two-cities-public-space-development-in-nigeria

[47] Xu F , Li J , Liang Y , et al . Associations of residential density with adolescents’ physical activity in a rapidly urbanizing area of mainland China. J Urban Health 2010;87:44–53. 10.1007/s11524-009-9409-9 19949994 PMC2821610

[48] Sallis JF , Glanz K . The role of built environments in physical activity, eating, and obesity in childhood. Future Child 2006;16:89–108. 10.1353/foc.2006.0009 16532660

[49] Giles-Corti B , Kelty SF , Zubrick SR , et al . Encouraging walking for transport and physical activity in children and adolescents: how important is the built environment. Sports Medicine 2009;39:995–1009. 10.2165/11319620-000000000-00000 19902982

[50] WHO . Global accelerated action for the health of adolescents (AA-HA!): guidance to support country implementation. Geneva WHO; 2017.

[51] Jalalkamali A , Doratli N . Public space behaviors and intentions: the role of gender through the window of culture, case of Kerman. Behav Sci (Basel) 2022;12:10. 10.3390/bs12100388 36285957 PMC9598707

[52] Van Hecke L , Verhoeven H , Clarys P , et al . Factors related with public open space use among adolescents: a study using GPS and accelerometers. Int J Health Geogr 2018;17:3. 10.1186/s12942-018-0123-2 29357871 PMC5778634

[53] Rutter H , Cavill N , Bauman A , et al . Systems approaches to global and national physical activity plans. Bull World Health Organ 2019;97:162–5. 10.2471/BLT.18.220533 30728623 PMC6357559

[54] Rutter H , Savona N , Glonti K , et al . The need for a complex systems model of evidence for public health. Lancet 2017;390:2602–4. 10.1016/S0140-6736(17)31267-9 28622953

[55] Popkin BM , Duffey K , Gordon-Larsen P . Environmental influences on food choice, physical activity and energy balance. Physiol Behav 2005;86:603–13. 10.1016/j.physbeh.2005.08.051 16246381

[56] Ioannidis JPA . Why most clinical research is not useful. PLoS Med 2016;13:e1002049. 10.1371/journal.pmed.1002049 27328301 PMC4915619

[57] World Health Organization . Engaging young people for health and sustainable development: strategic opportunities for the World Health Organization and partners; 2018.

[58] World Health Organization . Health for the world’s adolescents: a second chance in the second decade: summary. World Health Organization; 2014.

[59] Frerichs L , Ataga O , Corbie-Smith G , et al . Child and youth participatory interventions for addressing lifestyle-related childhood obesity: a systematic review. Obes Rev 2016;17:1276–86. 10.1111/obr.12468 27749992 PMC5555640

[60] Larsson I , Staland-Nyman C , Svedberg P , et al . Children and young people’s participation in developing interventions in health and well-being: a scoping review. BMC Health Serv Res 2018;18:507. 10.1186/s12913-018-3219-2 29954392 PMC6027768

[61] Anyon Y , Bender K , Kennedy H , et al . A systematic review of youth participatory action research (YPAR) in the United States: methodologies. Health Educ Behav 2018;45:865–78. 10.1177/1090198118769357 29749267

[62] Shamrova DP , Cummings CE . Participatory action research (PAR) with children and youth: an integrative review of methodology and PAR outcomes for participants, organizations, and communities. Child Youth Serv Rev 2017;81:400–12. 10.1016/j.childyouth.2017.08.022

[63] Horelli L , Kaaja M . Opportunities and constraints of "Internet-assisted urban planning" with young people. J Environ Psychol 2002;22:191–200. 10.1006/jevp.2001.0246

[64] Frank KI . The potential of youth participation in planning. J Plan Lit 2006;20:351–71. 10.1177/0885412205286016

[65] Cornish F , Breton N , Moreno-Tabarez U , et al . Participatory action research. Nat Rev Methods Primers 2023;3. 10.1038/s43586-023-00214-1

[66] Schoonenboom J , Johnson RB . How to construct a mixed methods research design. Köln Z Soziol 2017;69:107–31. 10.1007/s11577-017-0454-1 PMC5602001 28989188

[67] Bartlett L , Vavrus F . Rethinking case study research: a comparative approach. Taylor & Francis, 2016. 10.4324/9781315674889

[68] Beach D , Pedersen RB . Causal case study methods: foundations and guidelines for comparing, matching, and tracing. Ann Arbor, MI: University of Michigan Press, 2016. 10.3998/mpub.6576809

[69] ICPHR . Position paper 5: empowerment and Participatory health research. Baltimore ICfPHR; 2021.

[70] Wallerstein N . Empowerment to reduce health disparities. Scand J of Public Hlth 2002;30:72–7. 10.1080/140349402760232706 12227969

[71] Zimmerman MA . Psychological empowerment: issues and illustrations. American J of Comm Psychol 1995;23:581–99. 10.1007/BF02506983 8851341

[72] Zimmerman MA . Empowerment theory: psychological, organizational and community levels of analysis. In: Handbook of Community Psychology. Springer, 2000: 43–63. 10.1007/978-1-4615-4193-6

[73] Wang CC . Youth participation in Photovoice as a strategy for community. J Community Pract 2006;14:147–61. 10.1300/J125v14n01_09

[74] Amsden J , VanWynsberghe R . Community mapping as a research tool with youth. Action Research 2005;3:357–81. 10.1177/1476750305058487

[75] Carpiano RM . Come take a walk with me: the "go-along" interview as a novel method for studying the implications of place for health and well-being. Health Place 2009;15:263–72. 10.1016/j.healthplace.2008.05.003 18606557

[76] Vennix JAM , Akkermans HA , Rouwette E . Group model‐building to facilitate organizational change: an exploratory study. Syst Dyn Rev 1996;12:39–58. 10.1002/(SICI)1099-1727(199621)12:1<39::AID-SDR94>3.0.CO;2-K

[77] Luke DA , Stamatakis KA . Systems science methods in public health: dynamics, networks, and agents. Annu Rev Public Health 2012;33:357–76. 10.1146/annurev-publhealth-031210-101222 22224885 PMC3644212

[78] Hendricks G , Savona N , Aguiar A , et al . Adolescents' perspectives on the drivers of obesity using a group model building approach: a South African perspective. Int J Environ Res Public Health 2022;19:2160. 10.3390/ijerph19042160 35206348 PMC8871984

[79] O’Sullivan TL , Corneil W , Kuziemsky CE , et al . Use of the structured interview matrix to enhance community resilience through collaboration and inclusive engagement. Syst Res 2015;32:616–28. 10.1002/sres.2250

[80] Harputlugil T , Prins M , Gültekin AT , et al . Conceptual framework for potential implementations of multi criteria decision making (MCDM) methods for design quality assessment; 2011.

[81] Bernal JL , Cummins S , Gasparrini A . Interrupted time series regression for the evaluation of public health interventions: a tutorial. Int J Epidemiol 2017;46:348–55. 10.1093/ije/dyw098 27283160 PMC5407170

[82] Chelimsky E , Shadish W . An introduction to scientific realist evaluation. Evaluation for the 21st century: a handbook. Thousand Oaks CA, US: Sage Publications, Inc, 1997: 405–18. 10.4135/9781483348896

[83] Prins RG , Panter J , Heinen E , et al . Causal pathways linking environmental change with health behaviour change: natural experimental study of new transport infrastructure and cycling to work. Preventive Medicine 2016;87:175–82. 10.1016/j.ypmed.2016.02.042 26946367 PMC4893020

[84] Collins PH . Intersectionality’s definitional dilemmas. Annu Rev Sociol 2015;41:1–20. 10.1146/annurev-soc-073014-112142

[85] Ruger JP , Reiff M . A checklist for the conduct, reporting, and appraisal of microcosting studies in health care: protocol development. JMIR Res Protoc 2016;5:e195. 10.2196/resprot.6263 27707687 PMC5071616

[86] Reis RS , Salvo D , Ogilvie D , et al . Lancet physical activity series 2 executive C. Scaling up physical activity interventions worldwide: stepping up to larger and smarter approaches to get people moving. Lancet 2016;388:1337–48. 10.1016/S0140-6736(16)30728-0 27475273 PMC5193005

[87] Mittelmark MB . Unintended effects in settings-based health promotion. Scand J Public Health 2014;42:17–24. 10.1177/1403494814545108 25416569

[88] Gerritsen S , Harré S , Rees D , et al . Community group model building as a method for engaging participants and mobilising action in public health. Int J Environ Res Public Health 2020;17:3457. 10.3390/ijerph17103457 32429183 PMC7277214

[89] Luke DA , Harris JK . Network analysis in public health: history, methods, and applications. Annu Rev Public Health 2007;28:69–93. 10.1146/annurev.publhealth.28.021406.144132 17222078

[90] Emery M , Higgins L , Chazdon S , et al . Using ripple effect mapping to evaluate program impact: choosing or combining the methods that work best for you. JOE 2015;53. 10.34068/joe.53.02.28

[91] Chazdon S , Emery M , Hansen D , et al . A field guide to ripple effects mapping. University of Minnesota Libraries Publishing, 2017.

[92] Feick R , Robertson C . A multi-scale approach to exploring urban places in geotagged photographs. Comput Environ Urban Syst 2015;53:96–109. 10.1016/j.compenvurbsys.2013.11.006

[93] Pawlowski CS , Andersen HB , Troelsen J , et al . Children’s physical activity behavior during school recess: a pilot study using GPS, accelerometer, participant observation, and go-along interview. PLoS One 2016;11:e0148786. 10.1371/journal.pone.0148786 26859288 PMC4747537

[94] Pawlowski CS , Schmidt T , Nielsen JV , et al . Will the children use It?-A RE-AIM evaluation of a local public open space intervention involving children from a deprived neighbourhood. Eval Program Plann 2019;77:101706. 10.1016/j.evalprogplan.2019.101706 31472381

[95] Hofland ACL , Devilee J , van Kempen E , et al . Resident participation in neighbourhood audit tools - a scoping review. Eur J Public Health 2018;28:23–9. 10.1093/eurpub/ckx075 29346663 PMC5881759

[96] McKenzie TL . System for observing play and leisure activity in youth (SOPLAY). 2002: 2006. 10.1111/josh.13345 37340586

[97] McKenzie T , Cohen D . System for observing play and recreation in communities (SOPARC). Center for Population Health and Health Disparities (Ed) RAND 2006. 10.1123/jpah.3.s1.s208 28834508

[98] Manzano A . The craft of interviewing in realist evaluation. Evaluation 2016;22:342–60. 10.1177/1356389016638615

[99] Kern ML , Benson L , Steinberg EA , et al . The EPOCH measure of adolescent well-being. Psychol Assess 2016;28:586–97. 10.1037/pas0000201 26302102

[100] Zimmerman LA , Li M , Moreau C , et al . Measuring agency as a dimension of empowerment among young adolescents globally; findings from the global early adolescent study. SSM Popul Health 2019;8:100454. 10.1016/j.ssmph.2019.100454 31372490 PMC6660556

