# Supplementary material for: Youth-centred participatory action approach towards co-created implementation of socially and physically activating environmental interventions in Africa and Europe: the YoPA project study protocol
Source: BMJ Open. 2024 Feb 21;14(2):e084657. doi: 10.1136/bmjopen-2024-084657 (PMC10882351; doi:10.1136/bmjopen-2024-084657)
Supplement: Reviewer comments [file bmjopen-2024-084657.reviewer_comments.pdf]

PEER REVIEW HISTORY

BMJ Open publishes all reviews undertaken for accepted manuscripts. Reviewers are asked to complete a checklist review form (**http://bmjopen.bmj.com/site/about/resources/checklist.pdf**) and are provided with free text boxes to elaborate on their assessment. These free text comments are reproduced below.

ARTICLE DETAILS

**TITLE (PROVISIONAL)**	A Youth-centred Participatory Action approach towards co-created implementation of socially and physically activating environmental interventions in Africa and Europe: the YoPA project study protocol
**AUTHORS**	Chinapaw, Mai; Klaufus, Leonie; Oyeyemi, Adewale; Draper, Catherine; Palmeira, António L.; Silva, Marlene Nunes; Van Belle, Sara; Pawlowski, Charlotte; Schipperijn, Jasper; Altenburg, Teatske

This article was not externally reviewed at BMJ Open. Protocols that have been independently assessed prior to submission to BMJ Open are usually fast-tracked to publication on the grounds that further substantial changes will not be possible. This independent assessment will usually be external, independent review for both a substantial grant award from a non-commercial or government funder and ethics approval.
